# Bilateral bronchial stent deployment for palliative treatment of a compressive intrathoracic mass in a cat

**DOI:** 10.1177/2055116917753816

**Published:** 2018-02-09

**Authors:** Kieran Borgeat, Kerry Simpson, David Reese, Helen Wilson, Joanna Potter, Daniel Ogden

**Affiliations:** 1 Highcroft Veterinary Referrals, Bristol, UK; 2 Langford Vets, University of Bristol, UK; 3 VetCT Consultants in Telemedicine PTY LTD, Fremantle, Western Australia, Australia

## Abstract

**Case summary:**

Bronchial stents may be useful to relieve clinical signs of extraluminal compression. Herein we describe a case which, to our knowledge, is the first cat where bilateral bronchial stents have been used clinically. Respiratory signs of principal bronchial compression were alleviated after the stent procedure. Minor complications occurred, specifically: severe hypoxia during stent deployment; a transient, self-limiting postoperative pneumothorax possibly associated with ventilation-induced lung injury; bronchopneumonia (possibly pre-existing); and transient worsening of cough postoperatively. Stents were well- tolerated long- term. The cat was euthanased at 44 weeks post-stent procedure, owing to clinical signs of regurgitation, seemingly related to oesophageal dysfunction associated with tumour invasion.

**Relevance and novel information:**

In this case, it appeared that bronchial stents were feasible and the procedure was associated with long-term improvement in respiratory signs related to extraluminal bronchial compression.

## Introduction

Herein we describe a case of bronchial compression in a cat caused by a large heart base tumour, where dyspnoea was successfully treated by deploying bilateral bronchial stents.

## Case description

**Figure 1:**
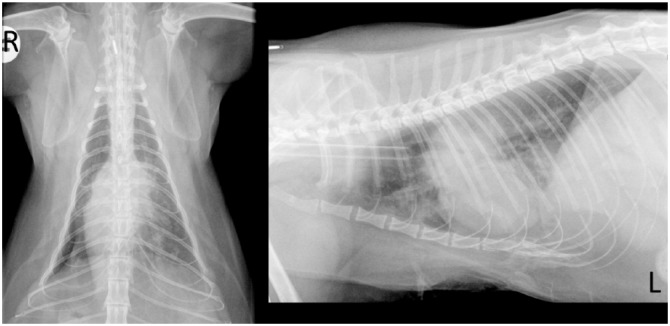
Dorsoventral and left lateral radiographs obtained at the primary veterinary clinic, demonstrating a soft tissue mass, cranial and dorsal to the cardiac silhouette, deviating the carina sternally. An alveolar pattern was present in the right middle and accessory lung lobes

A 5-year-old, female neutered, domestic shorthair cat (weight 4.65 kg; body condition score [BCS] 6/9) presented to a general practice for routine immunisation. A cough (2 weeks’ duration) was reported, with an in-clinic respiratory rate of 36 breaths/min and increased expiratory effort. One week later, thoracic radiography and bronchoalveolar lavage (BAL; blind technique) were performed. Radiography showed evidence of a soft-tissue mass, cranial and dorsal to the cardiac silhouette, deviating the carina sternally (Figure 1). A patchy alveolar pattern was present in the right middle and accessory lung lobes. There was no evidence of cardiomegaly or vascular distension. BAL yielded a profuse growth of *Pasteurella* species, with cytology supportive of neutrophilic inflammation.

**Figure 2:**
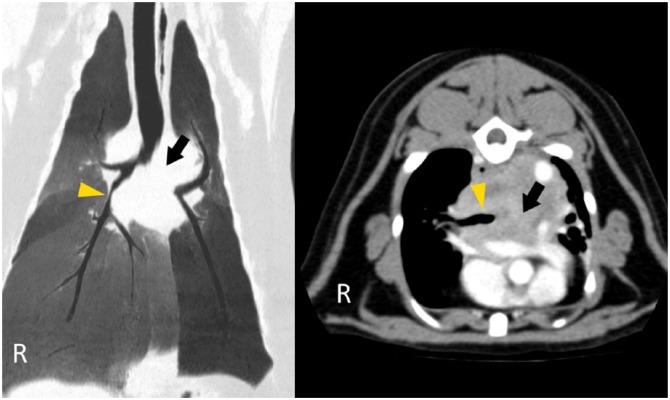
CT images of the heart base mass. The dorsal minimum intensity projection (left) shows compression of the right principal bronchus (arrowhead) and complete occlusion of the left principal bronchus (arrow). The transverse post-contrast section (right) illustrates the location and extent of the mass above the heart and great vessels (hyperintense with contrast) in addition to bilateral bronchial compression, as before

Upon referral, clinical findings were similar to those at initial assessment. Body weight had reduced to 4.29 kg (8% reduction over 14 days) with a BCS of 5/9. Rectal temperature was normal (38.6ºC). Complete blood count and serum biochemical profile showed no significant abnormalities; venous blood gas and electrolytes were within reference intervals. Serological tests for feline immunodeficiency virus and feline leukaemia virus (FeLV), and FeLV PCR were negative. A thoracic CT study (GE LightSpeed 16; GE Medical Systems) was elected and performed using helical acquisition, pre- and post-intravenous contrast medium administration. Images showed a large, contrast-enhancing mass at the heart base, which deviated the oesophagus dorsally and compressed the lumen of both principal bronchi, entirely obliterating the lumen of the left (Figure 2). The left caudal lung was reduced in size with diffuse soft tissue attenuation. The cranial mediastinal lymph nodes were enlarged. Ultrasound-guided needle aspirates of the mass yielded moderate numbers of cells with round nuclei, high nuclear to cytoplasmic ratio, highly stippled chromatin and a single, prominent nucleolus. There was no evidence of an infectious process. The most likely differentials were considered to be a mesothelioma or neuroendocrine tumour (chemodectoma most probable, given the location). CT findings of pulmonary infiltrates were considered likely to represent lobar consolidation with possible secondary infection, caused by bronchial obstruction and a lack of normal mucociliary clearance.

After consultation with the owners, palliative bronchial stenting was elected as the best option, owing to its minimally invasive nature and possibility of reducing the severity of dyspnoea. Radiation therapy was declined. Stent selection was based upon CT-acquired measurements of the bronchi: the length of the left principal bronchial compression was approximately 27.5 mm, the right 15.1 mm; diameter of the left bronchus prior to compression was 4.4 mm; comparable to the right side, which measured 4.6 mm. Two laser-cut self-expanding metallic stents were selected, measuring 5 mm diameter × 30 mm length, and were planned for deployment using a ‘kissing stents’ technique where the bronchial stents were deployed partially side by side in the trachea, to reduce the risk or rate of re-occlusion. Although commonly used in humans undergoing vascular interventions,^1,2^ this technique has also been performed for minimally invasive, palliative treatment for malignant bronchial obstruction in people.^3^

One week later, after intravenous premedication with butorphanol 0.2 mg/kg, dexamethasone 0.1 mg/kg and terbutaline 0.01 mg/kg (to reduce the risk of airway spasm and swelling during manipulations), the cat was induced using 0.5 mg/kg midazolam and 10 mg/kg ketamine (given slowly to effect) and intubated with a 4.5 mm endotracheal tube. Anaesthesia was maintained using a continuous rate infusion of alfaxalone (0.16 mg/kg/min) and manual intermittent positive pressure ventilation (IPPV) of 100% oxygen, using an Ayre’s T-piece. Perioperative antibiotics were administered (cefuroxime 20 mg/kg IV). Bronchoscopy (Olympus BF-P240 flexible video bronchoscope; Olympus Medical Systems) was performed in combination with fluoroscopic imaging (Arcadis Avantic C-arm; Siemens) to define the location of the carina (meeting point of the stents) and the location of the right middle and accessory lobar bronchi (RB2 and RB3) and the left caudal lobar bronchus (LB2). Complete occlusion of the left principal bronchus and compression of the right side were confirmed. Once these landmarks had been mapped on fluoroscopy, the cat was allowed a period of 5 mins pre-oxygenation (breathing 100% oxygen; Ayres’ T-piece, manual IPPV) before extubation for stenting, during which time SpO_2_ was consistently recorded at 92%, with arterial blood gas analysis measuring a PaO_2_ of only 100 mmHg, presumably associated with significant pulmonary compromise. Next, a 4 F Berenstein vascular catheter (4 F Berenstein 1 catheter 65 cm; Infiniti Medical) was advanced over a 0.035” angle-tipped hydrophilic ‘weasel’ guidewire (Infiniti Medical) into each of the principal bronchi. The weasel wires were removed and exchanged for more rigid 0.035” Rosen guidewires (0.035” 180 cm J-tip Rosen wire; Infiniti Medical), over which the Berenstein catheters were removed, leaving the guidewires in situ. Over each wire, the 5 mm × 30 mm self-expanding metallic stents (VetStent Uretha; Infiniti Medical) were simultaneously advanced within their deployment systems, and positioned so that the distal tips did not extend beyond the entrances to RB3/RB4 or LB2, but the proximal tips were located alongside one another, at the carina. Simultaneous deployment of the stents by two operators was performed, the delivery systems removed and the cat re-intubated. From extubation to re-intubation lasted 8 mins 47 s. Pulse oximetry readings reduced steadily; SpO_2_ of 49% was recorded at re-intubation. This increased to 90% within 5 mins. Arterial blood gas analysis was not performed at this point, so the accuracy of the pulse oximetry reading could not be confirmed.

**Figure 3:**
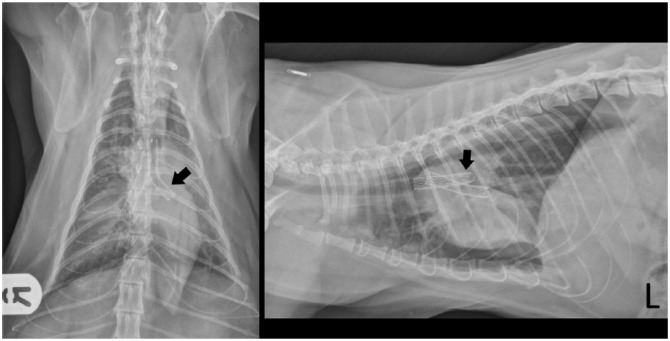
Postoperative dorsoventral and left lateral radiographic projections, obtained after needle thoracocentesis to drain a small pneumothorax. The bilateral bronchial stents are visible (left side indicated by arrow), as is some evidence of pulmonary pathology, which was considered most likely secondary to chronic bronchial obstruction. The position of the proximal portions of the stents alongside one another in the distal trachea is referred to as ‘kissing stents’ and reduces the risk of stent re-obstruction

Post-deployment bronchoscopy suggested appropriate stent position, confirmed by thoracic radiography. A small-volume pneumothorax was present: 60 ml of air was drained by needle thoracocentesis. Repeat radiographs showed resolution of pneumothorax and persistence of alveolar infiltrates, predominantly in the left lung (Figure 3). No recurrence of pneumothorax was present within 10 mins, so the cat was recovered in an oxygen-rich environment. Antibiotic treatment (cefuroxime 20 mg/kg IV q8h) was continued.

Six hours postoperatively, increased respiratory rate and effort were noted, and thoracic percussion suggested recurrence of pneumothorax. A further 100 ml of air was drained. No recurrence was noted overnight or the following morning, but coughing had worsened compared with preoperatively and appeared productive. Radiographs were not taken at this point. Nebulisation was performed every 4 h and oral antibiotics were administered: cephalexin 75 mg and clindamycin 25 mg q12hly. Improvement in demeanour and reduction in cough frequency were noted, and the cat was discharged 8 days post-procedure.

After discharge, the owner reported that the cat could not be medicated and would not eat tablets/capsules in food. However, at 14 days postoperatively the cat was well. Twenty-six days post-procedure, respiratory signs had worsened, with respiratory distress and coughing, in addition to reduced appetite and depressed demeanour. The cat returned for repeat thoracic radiography and bronchoscopy to exclude stent complications and perform BAL.

**Figure 4:**
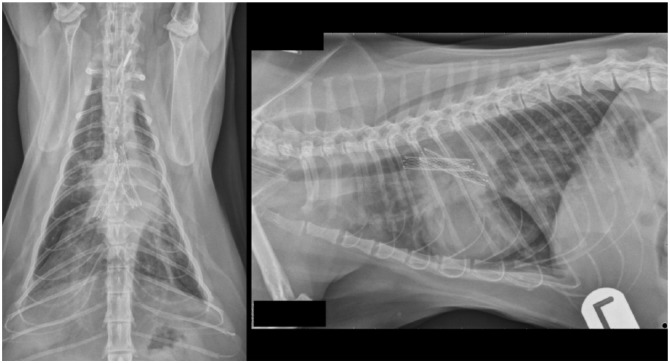
Dorsoventral and left lateral radiographic projections obtained 4 weeks after stent deployment, obtained to investigate worsening of respiratory signs. An interstitial-alveolar pattern is visible diffusely in the lung fields, worst in the right caudal lobe. Stent position and conformation was appropriate, with no evidence of migration or fracture

**Figure 5:**
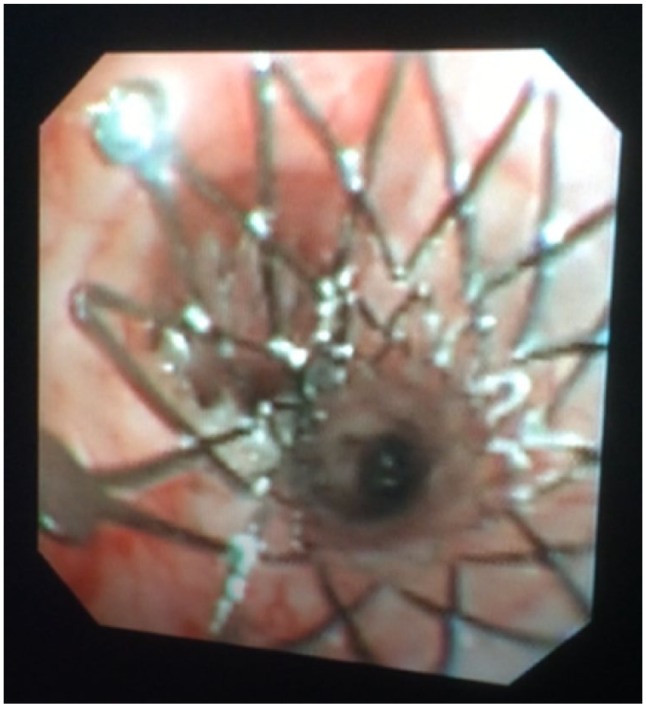
Still frame from bronchoscopy, showing no stent occlusion or malposition but a small amount of mucus accumulation. Bronchoalveolar lavage samples suggested that this was associated with bacterial infection

Radiography confirmed no stent migration or fracture. There was no evidence of pneumothorax or pleural effusion, but a bronchointerstitial pattern was present throughout the lungs, worst caudodorsally on the right (Figure 4). Bronchoscopy showed no evidence of stent occlusion (Figure 5), but excessive mucus production was evident, especially on the right. A neutrophilic exudate was present on BAL, with growth of a *Pasteurella* species, expressing the same resistance profile as previously. The cat was discharged the following day on oral pradofloxacin syrup (5 mg/kg PO q24h), which was tolerated well. Five days later, the cat had a normal demeanour and appetite. Cough was ongoing but less frequent (once or twice daily); respiratory rate had reduced to 30 breaths/min. Antibiotic treatment was discontinued after 30 days, with no respiratory signs aside from an intermittent, low-grade cough (two or three episodes of coughing per week, lasting <30 s each). At 27 weeks after the stent procedure, the cat was reported to have a good quality of life and normal activity levels. On examination, respiratory effort was normal with a mild increase in bronchovesicular sounds over the dorsal, left hemithorax. By 34 weeks postoperatively, regurgitation was evident 20–30 mins after eating, with an estimated frequency of one in three meals. The cat’s owner reported an otherwise bright demeanour but some loss of body condition (BCS 4/9). Repeat CT showed that the stents remained in an appropriate position, but an area of hyperintensity was visible in the wall of the oesophagus caudal to the heart base tumour, presumed to represent neoplastic infiltration. The cat was managed with postural feeding and dietary manipulation to reduce regurgitation frequency and severity. Owing to worsening clinical signs, the cat was euthanased 44 weeks post-stent, having exhibited no recurrence of dyspnoea or coughing.

## Discussion

Heart base tumours in cats have been described by several published case reports but appear to be rare in comparison with other cardiac neoplasia (namely myocardial lymphoma) and can affect adult cats of either sex.^4–12^ In contrast to dogs, pericardial effusion is not the primary presenting sign; respiratory signs predominate in cats, as identified in this case, where cough and dyspnoea were present.

In this case, the tumour type remained unknown because excisional biopsy was not performed and necropsy examination was not permitted. Cytology suggested a neuroendocrine origin (eg, chemodectoma), but histopathological review would be required to classify this neoplasm. Given that surgery to perform biopsy or debulking is invasive and associated with considerable risk of perioperative morbidity and mortality, it did not seem to be in the interests of this cat to pursue tissue sampling beyond needle aspiration to exclude lymphoma.

The only report of surgical or interventional treatment of a heart base tumour in the cat describes successful surgical resection, but with the subsequent sudden death of the cat 24 h later.^9^ Our case describes, to our knowledge, the first successful palliative treatment of a cat with clinical signs of a heart base tumour, also representing the first report of the clinical use of bronchial stents in this species.

Bronchial stents are becoming more frequently employed in the dog, where medical management of clinical signs associated with bronchial collapse or compression has proved unsuccessful,^13,14^ and reports of experimental bronchial stent use in the dog do exist.^15^ From our experience of this one feline case, we can state that bilateral bronchial stenting was feasible and well-tolerated by this cat and that clinical signs were ameliorated by the procedure. However, more experience is necessary to be able to make recommendations about the suitability of this treatment for cats in general and to provide a more accurate assessment of potential complications in this species.

Complications in this particular cat were mild and responded well to treatment. Hypoxia during interventional manipulations was expected, but the extent was estimated to be more severe than hoped, based on pulse oximetry readings. The degree of hypoxia may have been ambiguously represented, owing to reported inaccuracy of this technique in cats,^16,17^ but it seems likely to have been severe. However, with supportive ventilation after stent deployment, this appeared to resolve and the patient did not show any clinical signs of overt hypoxic brain injury postoperatively. The pneumothorax detected after bronchial stenting may have been associated with iatrogenic trauma or could have been associated with manual ventilation of diseased lungs (pre-existing bronchopneumonia). This appeared to be a self-limiting problem, and did not recur after needle thoracocentesis twice in the initial 6 h post-procedure; as such, stent-associated airway rupture seems unlikely. Infection with *Pasteurella* species was present before stent deployment and may not have been a direct complication of the procedure. However, clinical signs were responsive to antibiotic therapy and did not recur after cessation of a 30 day course, suggesting that the stents themselves were not acting as a nidus. It is possible that the signs of regurgitation, which were life-limiting in this case, were caused by a delayed manifestation of hypoxic injury to the brain’s white matter,^18^ but with evidence of tumour infiltration of the oesophagus on CT, it seems most likely that this was caused by neoplastic infiltration rather than being a consequence of the stent procedure.

## Conclusions

Bilateral bronchial stent deployment has proven to be feasible in a cat and helped to alleviate clinical signs of extraluminal bronchial compression in this case. The stents were well tolerated by this individual postoperatively. Mild but manageable complications of transient hypoxia, postoperative pneumothorax and bacterial bronchopneumonia did occur, but were controlled with appropriate supportive care and medical management. Ultimately, death was suspected to be related to neoplastic infiltration rather than signs of bronchial compression.
